# Supplementary material for: Patterns of Intron Gain and Loss in Fungi
Source: PLoS Biol. 2004 Nov 30;2(12):e422. doi: 10.1371/journal.pbio.0020422 (PMC532390; doi:10.1371/journal.pbio.0020422)
Supplement: Table S1 — Also available at http://genes.mit.edu/NielsenEtAl/. (4.3 MB ZIP). [file pbio.0020422.st001.zip › NielsenEtAl/html/263.html]

AN2417.1.NCU02531.1.MG09282.1.FG00363.1


```
 CLUSTAL W (1.82) Multiple Sequence Alignments - Introns Inserted


Sequence 1: NCU02531.1	954 aa
Sequence 2: MG09282.1	1016 aa
Sequence 3: FG00363.1	991 aa
Sequence 4: AN2417.1	967 aa
Alignment Length: 1058 aa
Number Identitical Residues: 180 aa
Alignment Score (without introns) 12480


MG09282.1 	MDDLLTPVSQVYTS-NKDKQPLLQEVTSSSRLIKNKSSATGSSLSS--YEDALEALKAEP
NCU02531.1	MNGLLTPISTSRLS-ASPNEPLLQEVGLRKETVKPNQSPGQRPLNS--PEEALEVLRHEP
FG00363.1 	MDDLLTPVSTTYLKPRKKTEPLFTEVKTSSPQPEPRR---ITAIST--ADDAVDILKNQP
AN2417.1  	MDKLITEIETIHHGQTPALTVREEKTKDFVSETKDATPTAESSSDSRSPAQILSTLKSTP
          	*: *:* :.               :.       :   ..   . .: :  : :. *:  *

MG09282.1 	DYDTLSSVLDFLLNGHTTKTQGAPCSVHQPNPLNSQIIQTLVTGIVPNYWQSLKDEAAKE
NCU02531.1	NYDQLVATLRFLS---HEDLAGSTFSIKEPSPMTAKLVQVLVSEIVPNYWTLLNED----
FG00363.1 	GYDSLISVLQFLT---STKPSSDNFSLSTPSPKSALIVHLLVSEIAPNYWTLLLEGSFDG
AN2417.1  	RREELSAAIFALDP-FNKRSSASSIDIRVPSPVSAQLLNALVTITIPDHWGSINAIN---
          	  : * :.:  *         .   .:  *.* .: ::: **:   *::*  :       

MG09282.1 	NQNLSQSQTKQAPLLQRLLSCLLNLTGVNVILTHLRGIVGELGTSTDETQRSMMSLNIAT
NCU02531.1	------SHETKSSALGLLLACLRSLTGVNAIIVRLRAWLQEAKSGDGKSKRPDIAINLAV
FG00363.1 	---VTQSAAALPRDAELFVACIQSLTGLNAVITQIRTLIQESRMGGREEKRTDLSLNANI
AN2417.1  	-----RTGTGDTKLRGALLRCLSSVSGINCLVTQLRSLIAQSRASSRQADASGSGRRIRH
          	      :    .     :: *: .::*:* ::.::*  : :   .  : . .  . .   

MG09282.1 	LLDLLCSLLHGHGALHRIWTNSSRTLGSESMLRPLTQEFVNVTGGGRIVSLAAEAERSAR
NCU02531.1	LLNLVCLLLKGNDSIRGIWKSATRTLNGNLNTRMLSQEMLSTFGSGRIIALTAEAIEHAK
FG00363.1 	LLSVLSAILDGHAAISAIWSASTKGIVNPALQRVQSQKLGSILTNGQIVSTSAEALEIIG
AN2417.1  	ILEVVSALLEPIDLVWRLYNDIDAVYGNATQKQVAWKELVSQLATGRLLSISAEATSLAG
          	:*.::. :*.    :  ::.       .    :   ::: .    *:::: :***     

MG09282.1 	KTSGET---NAWVASSKDYSKWLVRNIVNWAREDSLESPG--NPVSQLLTKALQLGHTD1
NCU02531.1	GHKAEIDVNNIWLANGPEYTKWLSQNIVKWQLTSPTSDET--KLCSELFLKALRLGHSD1
FG00363.1 	RDRERDE--TKWIADGLQYSKWMGNSIVSWVRSSPEADAM--TFASDLFQKSLSLHQSE1
AN2417.1  	ESDLPNK--ISWLGTGAQYASWLGANISHMASKLSVGNEEQWKAVASLTGRALSLGYTG1
          	      .    *:. . :*:.*:  .*       .  .  . .  :.*  ::* *  :  

MG09282.1 	LLVEYLVEGLLLSAVEKDVELFEKIFGNLPQHDQKRLLMAILK-YATNVFSSPEKS-KDE
NCU02531.1	TAMKCVLT-RLLIAQGADNERFGLLLGHVPRSEQRKVLFSVLKTFAEDYLNRLGTS-DTE
FG00363.1 	TLIKTVIDGLLLSKEGS-VQTFTRVALSQPRS-SKKVIHLLLHHLSRKYLNHLTLD-DDS
AN2417.1  	PLVREIYH--NLLVEQTHLEKYTALFDHLRWTEQLTVLDTIFRDIEKRCFSTESFSGNEK
          	  :. :     *       : :  :       ..  ::  :::      :.    ... .

MG09282.1 	AS--VVSAVAGLLRTIVKVDEKRRGILVGWLTSVSGGAAGEHVGIRRAAVGVLTEYRDDI
NCU02531.1	GSNKMISAVAGAISAITMNDPERIAYLVEWLT----------------------------
FG00363.1 	PDD-NVSAVAGLLKELALNDQFRRDTLIEWCASSSGAGLGDGVGIRRAVIATLSQDREAI
AN2417.1  	DLSEPIDDVAALCSALISKRASLESQLVDWLSDGQGGSIQTVSLRRAIIVN---------
          	  .. :. **.    :          *: * :. ....                      

MG09282.1 	TIVLEKTMALFGDNLYIKHAPLLQQEA1HAQILLLSAGYVHRLNPVKLAILVKSGVYLNA
NCU02531.1	--------------------------V1HAQVLLLSAGYVHKVSPLKLTLLMRSSPWLNA
FG00363.1 	ASVLEKSLAQFGDELYIRHSAILQQDV1HTQILLLAAGYMHRISPMKLTLLMRVGTYMTT
AN2417.1  	---------------------LAHQKA1NTQAILLAAGYLHRLNRDEVKSIGHTSVFLNA
          	                        ... ::* :**:***:*::.  ::  : : . ::.:

MG09282.1 	VSSRLSAPQTRARVLGMIVGEALSALTQGSGKALNFDMEILKTDEAKWYKSLVEVADTAG
NCU02531.1	ISNRLSSSNQRARFLGMVIGEGLSSLVDKGDKRLNFQVEEMEVPDGKWYKSLVEIQDTPG
FG00363.1 	ISNRIGATQSRAQFLGLVVGEALSSLIDDSKQRLNFKMEETDTEEAQQLKGLTKISDPVG
AN2417.1  	VSNRLAATSTRARFLGMIIGTAISQLIEEPGKAIKFDLEEMEGDEATWYLSLVNSSDKIG
          	:*.*:.:.. **:.**:::* .:* * :   : ::*.:*  .  :.    .*.:  *  *

MG09282.1 	SLEPLRQRVKVEL~PESKVGTQTQRPAEFVKPPKKTIPKPAHRATGFVIEEVS---ESEE
NCU02531.1	SWDPLLN--PQEL~TQKRTSSKT--------PSHKPAPS-QPRAQGFIIEELSD--ESLS
FG00363.1 	PTDPILSNIAIEP~TIQKRKHTTS-SKPAQKKAKQKKPIITEVKPKAIIEEIDSSDEDED
AN2417.1  	SLDSIRLP---KI1RAEARTSTTR-------PLGKSNQRTTKIVAVEEIDNSDE--EGED
          	. :.:      :    .     *           :             *:: ..  *. .

MG09282.1 	EDDNLVPYAKPDSDREDSDDDAELVSRNKPRAPV~YIRDLITYLRDSENYDKQELSLTTA
NCU02531.1	EQDDVVGYRKPDSDAEDSDDDPELVRRDKAKAPV~YIRDLIRYLRDTDDYDRQKLALVTA
FG00363.1 	EDEDLAPYFK-DSDPEDSDDDATLVQRNKIKPPV~YVRDLISYFRDSETYDKQLLALQTA
AN2417.1  	EDADLIPYEKPDEDPADEDDDPTLLQRNKPAAPV2YIRDLIIYLRDTENLDRYELALATA
          	*: ::  * *.*.*  *.***. *: *:*  .** *:**** *:**::  *:  *:* **

MG09282.1 	PVLIRRKANYGTEVVDHAEELAGLLVGIQDKFNLDDFESLRVQGMVSIIVAQPRKMGPWF
NCU02531.1	PTLIRRKANYGTEVTDHAEELASLLVGLQDKFEMDKFDDLRLQAMIAIVVTQPQLMGPWF
FG00363.1 	PVLIRRKANYGTEVSFHADELAGLLVGIQDKFEIENFQDLRVQGMVALVISQPKTMAPWL
AN2417.1  	PELIRRKAGFGTELAEHTEELGLVLVGLQEQSKFARFHELRLQSMIALIVSRPLKMARWF
          	* ******.:***:  *::**. :***:*:: ::  *..**:*.*:::::::*  *. *:

MG09282.1 	AKTFFDGDYSLSQRASVLTVLGLAAREIAGFEQSEFADS-------AYFPSKKLPEKVEQ
NCU02531.1	AKTFFDGDYSVSQRSLILTVLGLSAHELAGFETSEYAAA-------ASFPSKSLPEKIER
FG00363.1 	ARTFFEGDYSLYQRTSVLITLGLSARELAGFATSQYESA-------AAFPSKRLPEKMEQ
AN2417.1  	AAVFFDADLSQAQRSAVLIALGLSAREVAGVGADDAKAMGLPALSDTSFPSKRLPANLEA
          	* .**:.* *  **: :* .***:*:*:**.  .:    . .: :.: **** ** ::* 

MG09282.1 	FYLAQTGQNQVAGGSN-LKSLPPSAIDSIAQSITSGFLAPMVANAADEQTGPDLLKLSSF
NCU02531.1	LFISQGPSNAYQSSSSSLKPLPHNALESVATSITSSFLAPLSAAAADSATGPDVLKLSTF
FG00363.1 	LYIGPSGENKALPSSQ-LKALPANALDSISQSLTSEFLAPLAAEAADANTGPDVLKLQTF
AN2417.1  	FYAS-----------------PESPIASITRGIERASLQPIATEAADSVSGPKALKVRKF
          	:: .                 * ..: *:: .:    * *: : ***  :**. **: .*

MG09282.1 	ASRLQQRQGSGNSPLQ------------KGQKKAGVRAIPNTTAQLISASFFFPLTARFQ
NCU02531.1	KSRLPDYDDSNNAATSEEQHISNPKIKIKSRTKPRIRAIPNTTAQIISTSFFDPLTARFQ
FG00363.1 	TARYKS----------------------KTKAKPRVRAIPNTTAALLATSFFSPLAAHFQ
AN2417.1  	SSRMEVEKRR------------------QQREAHRQKSTVKDLYKVLSEGFFYPLTGRFE
          	 :*    .                    : :     ::  :    ::: .** **:.:*:

MG09282.1 	AALSSSTS~---RARTIVLQPELLTHLLRTLGILVHAAGPSTLSLPDMTAELWGLLLGSA
NCU02531.1	AALHSPVS~---RIRGIMFQPFLLALYLKTLALLLHAAGPSTLALPQMTGELWRLLLSTS
FG00363.1 	VALRS---~----AKPIILNHALLALYLQTLGIIVHAAGPSTLSLPQLTSELWDLLLG--
AN2417.1  	IMMMQFTS2STAPSHNPFFVPHLLTLFIQTLTIIISTSGPHTPFLPTLTHQTLTFLLSLH
          	  : .  : :::  :  .:   **:  ::** ::: ::** *  ** :* :   :**.  

MG09282.1 	VRARCAGDLGVTRAALFALLALLEVNGNE-EVRMRDICRELAREVVETQEWVSQVFENTR
NCU02531.1	VRAQAVGDVGITQAVLFGLLTLLDVN----EDRMRDVCQELGREVVETEGWVAGVFEGLR
FG00363.1 	VRVHVLGDLGATKGWLAAMASLLEVN----GGDLRRLCENQGREVMETRQWVSMVFERTR
AN2417.1  	ARPLSS-EPTILSALLSLFLAAIDLNLEFGTSGEERLVNEYAVQVMELREWVGEVFDQTP
          	.*     :     . *  : : :::* . .    . : .: . :*:* . **. **:   

MG09282.1 	GGDGEGGEENEVKLLAAGVLIRLRDAMERFRAFLIGDMIG---
NCU02531.1	GGD-EGREEERTKMLAAGVLVRLREGVEKYRLMMVGDLIGL--
FG00363.1 	GED--GGEENDVKMLAAGVLIRIGEAIEKYQALLMGDMIGFQ-
AN2417.1  	AVEDENDPRERVRTLAAGVMIKLGEVMERYQGRLMGVNSGFKY
          	. :...  .: .: *****:::: : :*:::  ::*   * .
```
